# The neurological adverse events of immune check point inhibitors in the treatment of cancer

**DOI:** 10.1007/s00415-026-13925-8

**Published:** 2026-06-06

**Authors:** Ahmad A. Toubasi, Thuraya N. Al-Sayegh

**Affiliations:** 1 Department of Neurology, Vanderbilt University Medical Center (VUMC), 1211 Medical Center Drive, 37232 Nashville, TN US; 2 Department of Internal Medicine, University of California San Francesco (UCSF), Fresno, CA US

**Keywords:** Immune checkpoint inhibitors, Neurological adverse effects, Cancer

## Abstract

**Background:**

Immune checkpoint inhibitors (ICIs) have revolutionized cancer treatment with significant improvements in survival rates. With the increase in ICI use in the clinical setting, several case reports and series have described neurological adverse events associated with it. We conducted this systematic review and meta-analysis to estimate the incidence of neurological adverse events among ICI clinical trials.

**Methods:**

We searched PubMed, Embase, Scopus, and the Cochrane Central Register of Controlled Trials (CENTRAL) on November 25, 2025, using ICI and clinical trial as keywords along with their related MeSH terms. The inclusion criteria involved clinical trials investigating the efficacy or safety of any ICI as the only systemic therapy among never treated patients with cancer. To eliminate the impact of other systemic therapies, trials that used ICIs as an adjuvant to another systemic treatment or included patients previously treated with any systemic therapies were excluded. The incidence and its 95% confidence interval (95%CI) were used as the effect measures in the analysis. Subgroup analyses were conducted based on the type of cancer and number of ICIs used in the trial.

**Results:**

We included a total of 8,826 patients with cancer from 31 clinical trials. The pooled incidence of neurological adverse events was 1.78 × 10^–3^% (95%CI 1.06 × 10^–3^%-2.78 × 10^–3^%). There was no difference (*p* = 0.481) in the incidence of neurological side effects between trials that used one ICI (1.83 × 10^–3^%; 95%CI 0.81 × 10^–3^%–3.22 × 10^–3^%) compared to those that used two ICIs (0.90 × 10^–3^%; 95%CI 0.01 × 10^–3^%–2.64 × 10^–3^%). There was also no significant difference (*p* > 0.622) in the incidence among patients with hepatocellular carcinoma (HCC) (1.20 × 10^–3^%; 95%CI 0.01 × 10^–3^%–3.67 × 10^–3^%), non-small cell lung carcinoma (NSCLC) (1.83 × 10^–3^%; 95%CI 0.01 × 10^–3^%–6.0 × 10^–3^%), and melanoma (1.16 × 10^–3^%; 95%CI 0.22 × 10^–3^%–2.71 × 10^–3^%). The most common neurological adverse events were peripheral neuropathy (1.34 × 10^–3^%; 95%CI 0.61 × 10^–3^%–2.3 × 10^–3^%), myositis (0.78 × 10^–3^%; 95%CI 0.29 × 10^–3^%–1.49 × 10^–3^%), aseptic meningitis (0.71 × 10^–3^%; 95%CI 0.25 × 10^–3^%–1.40 × 10^–3^%), autoimmune demyelinating polyneuropathy (0.66 × 10^–3^%; 95%CI 0.21 × 10^–3^%–1.32 × 10^–3^%), epilepsy (0.66 × 10^–3^%; 95%CI 0.21 × 10^–3^%–1.32 × 10^–3^%), and myasthenia gravis (0.66 × 10^–3^%; 95%CI 0.21 × 10^–3^%–1.32 × 10^–3^%).

**Conclusion:**

Taken all together, the incidence of ICI-related neurological adverse events among patients with cancer is low and estimated at 1 in 5000 patients. Individual data analysis of ICI clinical trials is needed to examine the factors associated with ICI-related neurological adverse events.

**Supplementary Information:**

The online version contains supplementary material available at 10.1007/s00415-026-13925-8.

## Introduction

Immune checkpoint inhibitors (ICIs) have revolutionized cancer treatment over the past 2 decades, transforming the landscape of cancer management [1]. Adaptive immune dysregulation plays a fundamental role in tumorigenesis. Cancer cells evade the immune system by utilizing the immune checkpoints such as programmed death protein 1 (PD-1), programmed death ligand 1 (PD-L1), cytotoxic T-lymphocyte associated protein 4 (CTLA-4), and lymphocyte activation gene 3 (LAG-3) used by healthy tissues to maintain self-tolerance. ICIs are monoclonal antibodies that target these checkpoints promoting immune surveillance and resulting in a robust anti-tumor immune response and destruction of malignant cells [2].

However, the impact of immune checkpoint inhibition is not limited to the tumor microenvironment. Immune checkpoints such as PD-1/PD-L1 and CTLA-4 are widely expressed across various healthy tissues. Consequently, downregulation of immune checkpoints can trigger the immune system to attack healthy tissues. The most frequently noted adverse events involve gastrointestinal, dermatologic, endocrine, and pulmonary systems. The increased use of ICIs over the past 2 decades helped to establish the characteristic features of these adverse events.

In contrast, neurological adverse events are more difficult to recognize and diagnose because their manifestations are non-specific [3]. The majority of available data in the literature comes from case reports and pharmacovigilance studies, which describe the onset of these conditions in a temporal relationship with ICI initiation [3]. It is also unclear how many of these symptoms have arisen due to prior/concurrent systemic cancer therapy such as chemotherapy or vascular endothelial growth factor (VEGF)-directed therapy because many of these medications are also associated with neurological side effects.

Determining whether these adverse events are related to ICIs is crucial for guiding their management strategies. Management begins with discontinuation of the ICI, followed by the initiation of immunosuppressive agents, such as corticosteroids, intravenous immunoglobulin, or mycophenolate mofetil, depending on the specific neurological adverse event. One of the vital steps in determining the etiology of an adverse event is estimating its incidence in isolation, independent of other systemic therapeutic agents.

To address these goals, we conducted a systematic review and meta-analysis to estimate the incidence of neurological adverse events in clinical trials assessing the safety of ICIs as a sole agent in the treatment of cancer patients who are treatment-naïve.

## Methods

### Registration and protocol

The protocol for this study was prospectively registered on the international prospective register of systematic reviews (PROSPERO; CRD420261290942). We followed the preferred reporting items for systematic reviews and meta-analyses (PRISMA) guidelines in conducting this study.

### Search strategy

On November 25, 2025, we searched PubMed, Embase, Scopus, and Cochrane Central Register of Controlled Trials (CENTRAL). The following search query was used (immune check-point inhibitors OR immune check-point inhibitor OR immune checkpoint inhibitors OR immune checkpoint inhibitor OR immune check point inhibitors OR immune check point inhibitor OR Ipilimumab OR Tremelimumab OR Yervoy OR Pidilizumab OR Opdivo OR Nivolumab OR Pembrolizumab OR Keytruda OR Tecentriq OR Atezolizumab OR Durvalumab OR Imfinzi OR Avelumab OR Bavencio OR cemiplimab OR Libtayo OR Jemperli OR Dostarlimab) AND (cancer OR neoplasm OR tumor OR tumour OR carcinoma OR metastasis OR leukemia OR lymphoma OR myeloma OR sarcoma OR melanoma) AND (clinical trials OR clinical trial OR trial).

The search results were downloaded and imported into Rayyan, a screening tool for systematic reviews and meta-analyses.

### Exposures and outcomes

The exposure of interest was any ICI including CTLA-4 inhibitors, PD-1 inhibitors, PD-L1 inhibitors, and LAG-3 inhibitors. The outcomes of interest were neurological adverse events including myelitis, myositis, myasthenia gravis, multiple sclerosis (MS), acute inflammatory demyelinating polyneuropathy (AIDP), chronic inflammatory demyelinating polyneuropathy (CIDP), peripheral neuropathy, autoimmune encephalitis, epilepsy, and aseptic meningitis between 2 and 60 months after the initiation of ICI. Myelitis was defined as inflammatory syndrome of the spinal cord causing bilateral motor and sensory dysfunction with MRI findings confirming the diagnosis. MS was defined as immune-mediated demyelinating disease of the CNS, characterized by dissemination in time and space. Autoimmune encephalitis was defined as subacute neuropsychiatric decline characterized by altered mental status, seizures, or psychiatric symptoms with magnetic resonance imaging (MRI) or cerebrospinal fluid (CSF) findings supporting the diagnosis. Epilepsy was defined as two or more unprovoked seizures more than 24 h apart. Aseptic meningitis was defined as headaches, fevers, neck stiffness, and photophobia with CSF analysis suggestive of inflammation but negative for bacterial, viral, fungal, or parasitic causes. Myositis was defined as proximal muscle weakness, elevated creatinine kinase, and electromyography (EMG), MRI or antibodies suggestive of the disease. Myasthenia gravis was defined as fatigable muscle weakness, with serological evidence or EMG findings supporting the diagnosis. AIDP was defined as monophasic demyelinating polyneuropathy with rapid ascending weakness with areflexia. CIDP was defined as chronic demyelinating neuropathy with progressive or relapsing weakness. Peripheral sensory neuropathy was defined as sensory or motor dysfunction of the peripheral nerves that do not fit the definition of AIDP or CIDP.

### Study selection

The inclusion criteria of our paper were (i) phase I, II, or III clinical trials that (ii) included treatment-naïve adults (age > 18 years old) with cancer and (iii) assessed the safety of ICIs as the sole treatment agent or combined with other local treatments. The exclusion criteria involved (i) trials among children, (ii) trials included previously treated patients with any systemic therapy other than ICIs, or (iii) trials that assessed the safety of ICIs in combination with other systemic therapies.

The study selection was performed in two stages; (i) title and abstract screening followed by (ii) full-text review. The selection process was done on Rayyan by AAT and TNA independently and any discrepancies were solved through discussion.

### Data extraction

The data extraction was performed on a prepared excel sheet. The data extraction variables included author information, the design of the trial, the trial phase, sample size, number of males/females, mean age, type and stage of cancer, the ICI used, and the data regarding the outcomes of interest.

The data extraction was conducted by AAT and TNA independently, and any discrepancies were resolved through discussion.

### Data analysis

The data analysis was done on MetaXL version 5.3 (EpiGear International, Australia). The incidence and its 95% confidence intervals (95%CIs) were used as the effect measures of interest [4]. The incidences of the neurological adverse events of interests were pooled using the random-effects model with double arcsine transformation. We first pooled the rates of all the neurological adverse events followed by analyzing the rate of each adverse event solely. We also conducted a subgroup analysis according to the number of ICIs used in the trial (one versus two) and the type of cancer. The subgroup analysis based on the type of cancer was done as feasible with at least three trials on the same type of cancer to conduct a subgroup analysis of that cancer. Across all analyses, *p* value < 0.050 was considered as statistically significant difference. The heterogeneity among the included studies was assessed using *I*^2^ and Cochrane *Q* statistics. Heterogeneity was considered significant if the *I*^2^ ≥ 50% and the *p* value < 0.050 while it was considered insignificant if *I*^2^ < 50% or the *p* value > 0.050.

## Results

### Search results

**Fig. 1:**
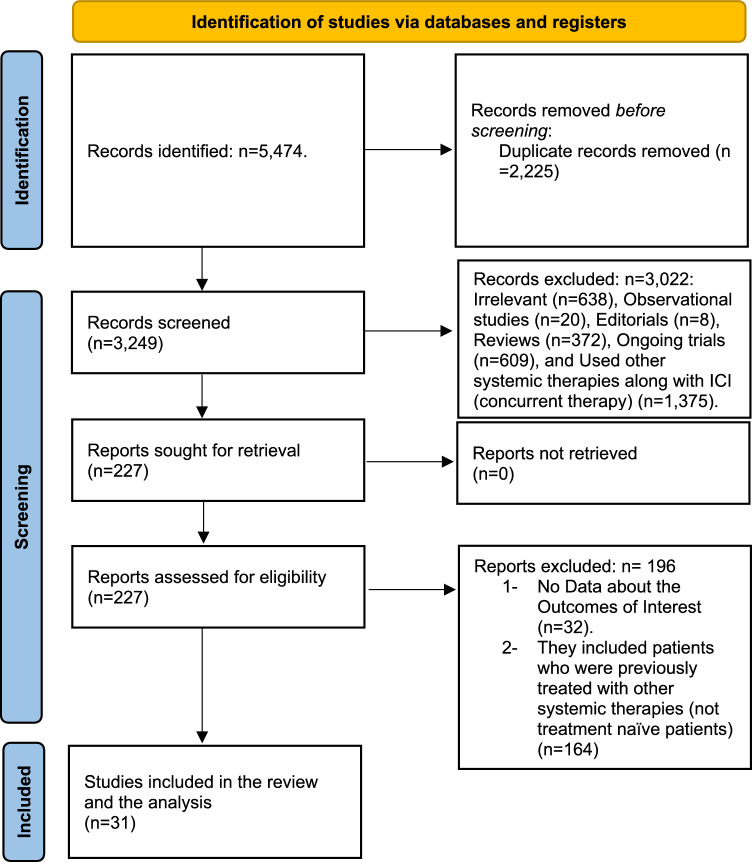
Preferred reporting items for systematic reviews and meta-analysis (PRISMA) flow chart. The figure represents the selection process of the included studies along with the number of excluded articles in each step and their reasons for exclusions (From [14])

Our search yielded 5474 articles, of which 2225 articles were duplicates. The remaining 3249 articles were screened using their titles and abstracts. Of these, 3022 articles were excluded as irrelevant, observational studies, editorials, reviews, ongoing trials, or trials that used other systemic therapies in combination with ICIs (concurrent therapy). The remaining 227 articles were screened using their full-text form and 196 articles were excluded because they did not report any data about the outcomes of interest or they included patients who were previously treated with other systemic therapies (not treatment-naïve patients). Finally, 31 articles were included in the final analysis. Supplementary references demonstrate the citations of the included papers. Figure 1 describes the study selection process along with reasons for exclusion.

### Characteristics of the included trials

The total number of the included patients was 8,825 from 31 articles. Of the included articles, 9.7% were phase I, 41.9% were phase II, and 48.4% were phase III clinical trials. Moreover, 22.6% included patients with NSCLC, 16.1% included patients with melanoma, and 9.6% included patients with HCC. The rest of the papers included patients with renal cell carcinoma, urothelial carcinoma, breast cancer, bladder cancer, gastric cancer, prostate cancer, ovarian cancer, and head and neck cancer. Additionally, 25.8% assessed two ICIs combined, and 61.3% assessed one ICI while the rest (12.9%) assessed one or two ICIs combined. The mean age of the included patients was 64.1 ± 11.5. The male to female ratio in the included trials was 2.27. Table 1 demonstrates the characteristics of the included studies.

**Table 1:** The characteristics of the included patients

Last author et al., year	Design	Phase	Sample size	Age of the included patients Mean (standard deviation)	Male to female ratio	Type of cancer
Van Dijk et al., 2020	Multi-center single arm	II	24	65 (8)	3	Stage III urothelial cancer
Yau et al., 2022	Multi-center randomized	III	743	65 (4)	5.63	Stage I–III HCC
Hasegawa et al., 2022	Multi-center single arm	I	31	69 (10)	2.1	Stage I–III gastric cancer
Kuemmel et al., 2025	Multi-center single arm	II	43	57 (14)	N/A	Stage I–III breast cancer
Grivas et al., 2024	Multi-center randomized	I	40	57 (10)	2.08	Stage I–III muscle invasive bladder cancer
Chaft et al., 2022	Multi-center single arm	II	181	65 (9)	0.95	Stage IB–IIIB NSCLC
Becker et al., 2023	Multi-center open label	II	118	71 (13)	1.95	Stage I–IV Merkel cell carcinoma
Altorki et al., 2021	Single-center randomized	II	30	71 (2)	1.14	Stage I–III NSCLC
Piganta et al., 2023	Multi-center randomized	III	651	60 (14)	N/A	Stage III–IV ovarian cancer
Powles et al., 2022	Multi-center randomized	III	496	60 (4)	2.33	Stage I–IV RCC
Eichhorn et al., 2025	Single-center single arm	II	29	61.3 (11)	0.81	Stage II–III NSCLC
Shao et al., 2025	Single-center single arm	II	30	66.9 (10.3)	14	Stage II–III HCC
Rusch et al., 2023	Multi-center single arm	II	181	65 (9)	1.06	Stage I–III NSCLC
Albiges et al., 2020	Multi-center randomized	III	550	62 (15)	3.01	Stage IV RCC
Ascierto et al., 2020	Multi-center randomized	III	906	55 (5)	1.39	Stage III–IV melanoma
Breukers et al., 2025	Multi-center randomized	II	50	76 (15)	4.56	Stage I–IV SCC
Hui et al., 2017	Multi-center randomized	I	101	68 (13)	1.46	Stage IV NSCLC
Beer et al., 2017	Multi-center randomized	III	400	70 (12)	N/A	Stage I–IV prostate cancer
Wolchok et al., 2024	Multi-center randomized	III	941	61 (17)	1.82	Stage III–IV melanoma
Shaverdashvili et al., 2023	Multi-center single arm	II	47	79 (10)	1.35	Stage III–IV NSCLC
Eggermont et al., 2015	Multi-center randomized	III	475	51 (16)	1.65	Stage III Melanoma
Allaf et al., 2024	Multi-center randomized	III	381	60 (14)	2.37	Stage I–IV RCC
Kim et al., 2024	Multi-center randomized	II	48	61 (14)	8.6	Stage II–IVa HNSCC
Rizvi et al., 2020	Multi-center randomized	III	326	64 (13)	2.43	Stage IV NSCLC
Long et al., 2024	Multi-center randomized	III	656	60 (14)	1.95	Stage III–IV melanoma
Ascierto et al., 2018	Multi-center randomized	III	210	64 (17)	1.36	Stage III–IV melanoma
Bergmann et al., 2025	Multi-center randomized	III	157	61 (18)	2.49	Stage III–IV RCC
Zandberg et al., 2025	Multi-center randomized	II	28	72.5 (13)	0.04	Stage III–IV SCC
Vasudev et al., 2023	Multi-center randomized	II	192	62 (13)	3.47	Stage III–IV RCC
Yau et al., 2025	Multi-center randomized	III	335	65 (3)	4.23	Stage I–IV HCC
Motzer et al., 2019	Multi-center randomized	III	425	62 (15)	2.83	Stage III–IV RCC

*HCC*   hepatocellular carcinoma, *HNSCC*   head and neck squamous cell carcinoma, *NSCLC*   non-small cell lung cancer, *RCC*   renal cell carcinoma, and *SCC*  squamous cell carcinoma

### Incidence of all neurological adverse effects

A total of 31 articles were included in the model that assessed the incidence of all neurological adverse effects. The model found that the pooled incidence of neurological adverse events was 1.78*10^–3^% (95%CI 1.06*10^–3^%–2.78*10^–3^%); the heterogeneity of the model was significant (*I*^2^ = 35.4%, *p* = 0.028).

Subgroup analysis of trials that assessed one ICI included 19 articles and found an incidence of 1.83*10^–3^% (95%CI 0.81*10^–3^%–3.22*10^–3^%); the heterogeneity of the model was not significant (*I*^2^ = 25.6%, *p* = 0.149). The model that pooled trials that assessed two ICIs combined included eight articles and found an incidence of 0.90*10^–3^% (95%CI 0.03*10^–3^%–2.64*10^–3^%); the heterogeneity of the model was not significant (*I*^2^ = 2.3%, *p* = 0.412). There was no significant difference in the incidence between the two groups (*p* = 0.481).

Subgroup analysis according to the type of cancer demonstrated no significant difference (*p* > 0.622) in the incidence of neurological adverse effects between patients with HCC (*n* = 3 trials, incidence = 1.20*10^–3^%; 95%CI 0.01*10^–3^%–3.67*10^–3^%), patients with NSCLC (*n* = 7 trials, incidence = 1.83*10^–3^%; 95%CI 0.01*10^–3^%–6.04*10^–3^%), and melanoma (*n* = 5 trials, incidence = 1.16*10^–3^%; 95%CI 0.22*10^–3^%–2.71*10^–3^%). The heterogeneity of the models was not significant (*I*^2^ < 20%, *p* > 0.289).

### Incidence of each type of neurological adverse effect

Among all the included trials, peripheral neuropathy had the highest incidence with a rate of 1.34*10^–3^% (95%CI 0.61*10^–3^%–2.3*10^–3^%); the heterogeneity of the model was not significant (*I*^2^ = 10.0%, *p* = 0.308). Subgroup analysis demonstrated no difference in the incidence of peripheral neuropathy between trials that assessed one ICI compared to those that used two ICIs combined (Table 2). The heterogeneity of both models was not significant (*I*^2^ < 19.4%, *p *> 0.289). Analysis based on the cancer type showed no difference in the incidence of peripheral neuropathy between trials among patients with HCC, NSCLC, and melanoma (Table 2). The heterogeneity of the models was not significant (*I*^2^ < 19.4%, *p* > 0.289).

**Table 2:** The incidence of adverse neurological events across different subgroups expressed as incidence*10^–3^% (95% confidence interval)

Adverse event	One ICI	Two ICIs combined	HCC	NSCLC	Melanoma
Peripheral neuropathy	1.02 (0.28–2.08)	0.91 (0.01–2.70)	1.45 (0.01–4.89)	1.64 (0.01–4.82)	1.16 (0.22–2.71)
Myositis	0.91 (0.23–1.97)	0.69 (0.01–1.95)	0.53 (0.01–2.32)	1.64 (0.01–4.82)	0.37 (0.01–1.19)
Aseptic meningitis	0.78 (0.16–1.78)	0.69 (0.01–1.95)	0.53 (0.01–2.32)	1.64 (0.01–4.82)	0.37 (0.01–1.19)
AIDP	0.69 (0.12–1.65)	0.69 (0.01–1.95)	0.53 (0.01–2.32)	1.64 (0.01–4.82)	0.51 (0.01–1.42)
Epilepsy	0.69 (0.12–1.65)	0.69 (0.01–1.95)	0.53 (0.01–2.32)	1.64 (0.01–4.82)	0.37 (0.01–1.19)
Myasthenia gravis	0.69 (0.12–1.65)	0.69 (0.01–1.95)	0.53 (0.01–2.32)	1.64 (0.01–4.82)	0.37 (0.01–1.19)

*AIDP*  autoimmune demyelinating polyneuropathy, *HCC*   hepatocellular carcinoma, *ICI* immune checkpoint inhibitor, *NSCLC*   non-small cell lung cancer

The pooled incidence of myositis was 0.78 *10^–3^% (95%CI 0.29*10^–3^%–1.49 *10^–3^%); the heterogeneity of the model was not significant (*I*^2^ = 0.0%, *p* = 0.993). There was no difference in the incidence of myositis between trials that used one ICI compared to those that used two ICIs (Table 2). The heterogeneity of both models was not significant (*I*^2^ = 0.0%, *p* > 0.880). There was no significant difference in the incidence between patients with HCC, NSCLC, and melanoma (Table 2). The heterogeneity of the models was not significant (*I*^2^ = 0.0%, *p* > 0.725).

The pooled incidence of aseptic meningitis was 0.71*10^–3^% (95%CI 0.25*10^–3^%–1.40*10^–3^%); the model had insignificant heterogeneity (*I*^2^ = 0.0%, *p* = 1.000). There was no difference in the incidence of aseptic meningitis between trials that assessed one ICI compared to those that assessed two ICIs (Table 2); the heterogeneity of both models was not significant (*I*^2^ = 0.0%, *p* > 0.985). There was also no difference between trials among patients with HCC, NSCLC, and melanoma (Table 2). The heterogeneity of the models was not significant (*I*^2^ = 0.0%, *p* > 0.725).

The pooled incidence of autoimmune demyelinating polyneuropathy was 0.66*10^–3^% (95%CI 0.21*10^–3^%–1.32*10^–3^%); the heterogeneity of the model was not significant (*I*^2^ = 0.0%, *p* = 1.000). There was no difference in the incidence of AIDP between trials that assessed one ICI compared to those that assessed two ICIs (Table 2). There was also no difference in the incidence between patients with HCC, NSCLC, and melanoma (Table 2). The heterogeneity of the models was not significant (*I*^2^ = 0.0%, *p* > 0.532).

The pooled incidence of epilepsy was 0.66 *10^–3^% (95%CI 0.21*10^–3^%–1.32*10^–3^%); the heterogeneity of the model was not significant (*I*^2^ = 0.0%, *p* = 1.000). There was no difference in the incidence of epilepsy between trials that assessed one ICI compared to those that assessed two ICIs (Table 2). The heterogeneity of the models was not significant (*I*^2^ = 0.0%, *p* > 0.982). There was also no significant difference in the incidence of epilepsy between trials among patients with HCC, NSCLC, and melanoma (Table 2). The heterogeneity of the models was not significant (*I*^2^ = 0.0%, *p* > 0.725).

The pooled incidence of myasthenia gravis was 0.66*10^–3^% (95%CI 0.21*10^–3^%–1.32*10^–3^%). The incidence of myasthenia gravis was not different between trials that assessed one ICI compared to those that assessed two ICIs (Table 2). The heterogeneity of the models was not significant (*I*^2^ = 0.0%, *p* > 0.725). The incidence was not different between patients with HCC, NSCLC, and melanoma (Table 2). The heterogeneity of the models was not significant (*I*^2^ = 0.0%, *p* > 0.983).

There were no cases of CIDP, MS, or autoimmune encephalitis reported in the included trials.

## Discussion

Our systematic review and meta-analysis aimed to estimate the incidence of neurological adverse events due to ICI among treatment-naïve patients with cancer during clinical trials that investigated their use as a single-agent therapy. Our analysis revealed three key findings. First, the incidence of ICI-related neurological adverse events in clinical trials is low and is estimated at one adverse event per 5000 treated patients. Second, the most commonly reported neurological adverse events were peripheral neuropathy followed by myositis, aseptic meningitis, AIDP, epilepsy, and myasthenia gravis. Third, there was no difference in the incidence of the reported adverse events based on the number of ICIs used or the type of cancer.

The potential of ICI has been hindered by their immune-related adverse events [5]. A previous systematic review and meta-analysis that was done by Amaud-Coffin and collaborators to investigate the incidence of immune-related adverse events of ICIs among clinical trials demonstrated that grade ≥ 3 immune-related adverse events were estimated at 14% among patients treated with PD-1/PD-L1 inhibitors and 34% among patients treated with CTLA-4 inhibitors [6]. However, neurological immune adverse events represent a minority of these. A previous systematic review conducted by Cuzzubbo and collaborators [7] that reviewed the literature reporting neurological adverse events associated with ICIs estimated that the incidence of grade ≥ 3 adverse events in the clinical trials was less than 0.7% with the majority of them being headaches. In contrast, our study found a lower incidence of neurological adverse events, estimated at one per 5000 patients. The large difference in the estimated incidence stems from two major methodological differences. First, we did not include non-specific neurological adverse events such as headaches. Second, we had more strict exclusion criteria compared to the study done by Cuzzubbo and collaborators [7] as we excluded trials that included patients previously treated with systemic therapies other than ICI and trials that tested other systemic therapies in combination with ICIs. We followed these exclusion criteria to limit the impact of other chemo- and immunotherapies that are known to cause similar neurological adverse events.

In terms of the most commonly reported neurological adverse events, we found that peripheral neuropathy and myositis had the highest incidence. A previous systematic review and meta-analysis of published case reports in the literature found 428 case reports with the most commonly reported adverse events being myositis and peripheral neuropathy [8] which is consistent with our findings. This highlights that the general epidemiological scheme of reporting between the clinical trials and real-world data is similar. It is important to highlight that case reports of myelitis, encephalitis, and multiple sclerosis were found in the aforementioned review but not in our analysis. Two main hypotheses might explain these differences. One possible explanation is that these syndromes may be caused by previous/concurrent exposure to other systemic therapies, or simply cancer progression [9–12]. It is also possible that these cases needed more time or were more latent in their presentation compared to the follow-up duration of the clinical trials [13].

We did not find a difference in the incidence of neurological adverse events based on the type of cancer. These findings are somewhat similar but also different from the work done by Marini et al. using the case reports published in the literature [8]. Similar to our findings, Marini and colleagues [8] found that there was no significant difference in the overall neurological adverse events incidence across different cancer types. However, they found that only peripheral neuropathy was more common among patients with melanoma compared to other cancer types [8]. Again, these findings should consider the caveat that the study included cases previously treated with other systemic therapies [8]. Another explanation for the lack of significance in our findings is the small number of events found in the trials which could limit the power of the analysis.

Surprisingly, we also did not find a significant difference in the incidence of the neurological adverse events between trials that assessed one ICI compared to the trials that assessed two ICIs combined. A previous single center retrospective cohort study of 1834 patients who received ICI demonstrated that grade ≥ 3 neurological adverse events were reported in 1.5% among patients treated with single ICI compared to 3% among patients treated with dual agents combined [5]. However, our findings raise the question whether more hits on the same pathway (immune tolerance) would increase the frequency of ICI-related neurological adverse events, or whether once a hit is tolerated by the autoimmune system, a concurrent hit on the same pathway will have no effect.

Before concluding, few limitations should be acknowledged. First, the low number of events limited the power of the subgroup analysis. Second, we did not assess the quality of the clinical trials as the aim of the paper was neither focused on the safety nor the efficacy of ICI, which has already been established, but rather on better understanding the neurological adverse events associated with ICI. Third, we were not able to conduct a subgroup analysis for cancers other than NSCLC, HCC, and melanoma because the rest of the cancers were evaluated by less than three trials. Future meta-analyses with a larger number of trials should aim to perform such subgroup analyses. Finally, we did not use the previously proposed definitions of ICI-related neurological adverse events like the one proposed by Guidon et al. 2021 which would categorize these events. However, we preferred to isolate each disease separately in the analysis as the clinical management of each one of these adverse events might be different. Moreover, the included trials did not use any of the proposed definitions of ICI-related neurological adverse events which limits our ability to use these definitions. Future clinical trials are recommended to use such definitions to unify the reporting of these adverse events across clinical trials.

In conclusion, after removing the effect of other systemic chemo- and immunotherapies, the incidence of neurological adverse events due to ICI is much lower than previously reported. Surprisingly, the incidence of neurological adverse events was not different based on the tumor type or the number of ICIs used. A more accurate estimation of the incidence of ICI-related neurological adverse events and a deeper understanding of its pathophysiology are needed.

## Supplementary Information

Below is the link to the electronic supplementary material.

Supplementary file1 (DOCX 107 KB)

Supplementary file2 (DOCX 269 KB)

## Data Availability

The data is available from the corresponding author upon reasonable request.
