# Supplementary material for: The neurological adverse events of immune check point inhibitors in the treatment of cancer
Source: J Neurol. 2026 Jun 6;273(7):373. doi: 10.1007/s00415-026-13925-8 (PMC13242459; doi:10.1007/s00415-026-13925-8)
Supplement: Supplementary file 1 — Supplementary file1 (DOCX 107 KB) [file 415_2026_13925_MOESM1_ESM.docx]

**Supplementary references^1-30^**

1. Stockem CF, van Dorp J, van Dijk N, et al. Final clinical analysis of pre-operative ipilimumab and nivolumab in locally advanced urothelial cancer and exploration of tumor-draining lymph node composition: The NABUCCO trial. *European journal of cancer (Oxford, England : 1990).* 2025;229:115731.

2. Yau T, Park JW, Finn RS, et al. Nivolumab versus sorafenib in advanced hepatocellular carcinoma (CheckMate 459): a randomised, multicentre, open-label, phase 3 trial. *The Lancet Oncology.* 2022;23(1):77-90.

3. Hasegawa H, Shitara K, Takiguchi S, et al. A multicenter, open-label, single-arm phase I trial of neoadjuvant nivolumab monotherapy for resectable gastric cancer. *Gastric cancer : official journal of the International Gastric Cancer Association and the Japanese Gastric Cancer Association.* 2022;25(3):619-628.

4. Kuemmel S, Graeser M, Schmid P, et al. Chemotherapy-free neoadjuvant pembrolizumab combined with trastuzumab and pertuzumab in HER2-enriched early breast cancer (WSG-KEYRICHED-1): a single-arm, phase 2 trial. *The Lancet Oncology.* 2025;26(5):629-640.

5. Grivas P, Koshkin VS, Chu X, et al. PrECOG PrE0807: A Phase 1b Feasibility Trial of Neoadjuvant Nivolumab Without and with Lirilumab in Patients with Muscle-invasive Bladder Cancer Ineligible for or Refusing Cisplatin-based Neoadjuvant Chemotherapy. *European urology oncology.* 2024;7(4):914-922.

6. Chaft JE, Oezkan F, Kris MG, et al. Neoadjuvant atezolizumab for resectable non-small cell lung cancer: an open-label, single-arm phase II trial. *Nature Medicine.* 2022;28(10):2155-2161.

7. Becker JC, Ugurel S, Leiter U, et al. Adjuvant immunotherapy with nivolumab versus observation in completely resected Merkel cell carcinoma (ADMEC-O): disease-free survival results from a randomised, open-label, phase 2 trial. *The Lancet.* 2023;402(10404):798-808.

8. Altorki NK, McGraw TE, Borczuk AC, et al. Neoadjuvant durvalumab with or without stereotactic body radiotherapy in patients with early-stage non-small-cell lung cancer: a single-centre, randomised phase 2 trial. *The Lancet Oncology.* 2021;22(6):824-835.

9. Pignata S, Bookman M, Sehouli J, et al. Overall survival and patient-reported outcome results from the placebo-controlled randomized phase III IMagyn050/GOG 3015/ENGOT-OV39 trial of atezolizumab for newly diagnosed stage III/IV ovarian cancer. *Gynecologic oncology.* 2023;177:20-31.

10. Powles T, Tomczak P, Park SH, et al. Pembrolizumab versus placebo as post-nephrectomy adjuvant therapy for clear cell renal cell carcinoma (KEYNOTE-564): 30-month follow-up analysis of a multicentre, randomised, double-blind, placebo-controlled, phase 3 trial. *The Lancet Oncology.* 2022;23(9):1133-1144.

11. Eichhorn ME, Niedermaier B, Charoentong P, et al. Neoadjuvant anti-programmed death-1 immunotherapy by pembrolizumab in resectable non-small cell lung cancer: results of the NEOMUN trial. *Journal for immunotherapy of cancer.* 2025;13(8).

12. Rusch VW, Nicholas A, Patterson GA, et al. Surgical results of the Lung Cancer Mutation Consortium 3 trial: A phase II multicenter single-arm study to investigate the efficacy and safety of atezolizumab as neoadjuvant therapy in patients with stages IB-select IIIB resectable non-small cell lung cancer. *The Journal of thoracic and cardiovascular surgery.* 2023;165(3):828-839.e825.

13. Albiges L, Tannir NM, Burotto M, et al. Nivolumab plus ipilimumab versus sunitinib for first-line treatment of advanced renal cell carcinoma: extended 4-year follow-up of the phase III CheckMate 214 trial. *ESMO open.* 2020;5(6):e001079.

14. Ascierto PA, Del Vecchio M, Mandalá M, et al. Adjuvant nivolumab versus ipilimumab in resected stage IIIB-C and stage IV melanoma (CheckMate 238): 4-year results from a multicentre, double-blind, randomised, controlled, phase 3 trial. *The Lancet Oncology.* 2020;21(11):1465-1477.

15. Breukers SE, Traets JJH, van Dijk SW, et al. Neoadjuvant ipilimumab and nivolumab in resectable cutaneous squamous cell carcinoma: a randomized phase 2 trial. *Nature Medicine.* 2025;31(12):4055-4064.

16. Hui R, Garon EB, Goldman JW, et al. Pembrolizumab as first-line therapy for patients with PD-L1-positive advanced non-small cell lung cancer: a phase 1 trial. *Annals of oncology : official journal of the European Society for Medical Oncology.* 2017;28(4):874-881.

17. Beer TM, Kwon ED, Drake CG, et al. Randomized, Double-Blind, Phase III Trial of Ipilimumab Versus Placebo in Asymptomatic or Minimally Symptomatic Patients With Metastatic Chemotherapy-Naive Castration-Resistant Prostate Cancer. 2017;35(1):40-47.

18. Wolchok JD, Chiarion-Sileni V, Rutkowski P, et al. Final, 10-Year Outcomes with Nivolumab plus Ipilimumab in Advanced Melanoma. *The New England journal of medicine.* 2025;392(1):11-22.

19. Shaverdashvili K, Reyes V, Wang H, et al. A phase II clinical trial evaluating the safety and efficacy of durvalumab as first line therapy in advanced and metastatic non-small cell lung cancer patients with Eastern Cooperative Oncology Group performance status of 2. *EClinicalMedicine.* 2023;66:102317.

20. Eggermont AM, Chiarion-Sileni V, Grob JJ, et al. Adjuvant ipilimumab versus placebo after complete resection of high-risk stage III melanoma (EORTC 18071): a randomised, double-blind, phase 3 trial. *The Lancet Oncology.* 2015;16(5):522-530.

21. Allaf M, Kim SE, Harshman LC, et al. LBA67 Phase III randomized study comparing perioperative nivolumab (nivo) versus observation in patients (Pts) with renal cell carcinoma (RCC) undergoing nephrectomy (PROSPER, ECOG-ACRIN EA8143), a National Clinical Trials Network trial. *Annals of Oncology.* 2022;33:S1432-S1433.

22. Kim CG, Hong MH, Kim D, et al. A Phase II Open-Label Randomized Clinical Trial of Preoperative Durvalumab or Durvalumab plus Tremelimumab in Resectable Head and Neck Squamous Cell Carcinoma. *Clinical cancer research : an official journal of the American Association for Cancer Research.* 2024;30(10):2097-2110.

23. Rizvi NA, Cho BC, Reinmuth N, et al. Durvalumab With or Without Tremelimumab vs Standard Chemotherapy in First-line Treatment of Metastatic Non–Small Cell Lung Cancer: The MYSTIC Phase 3 Randomized Clinical Trial. *JAMA Oncology.* 2020;6(5):661-674.

24. Long GV, Lipson EJ, Hodi FS, et al. First-Line Nivolumab Plus Relatlimab Versus Nivolumab Plus Ipilimumab in Advanced Melanoma: An Indirect Treatment Comparison Using RELATIVITY-047 and CheckMate 067 Trial Data. 2024;42(33):3926-3934.

25. Ascierto PA, Long GV, Robert C, et al. Survival Outcomes in Patients With Previously Untreated BRAF Wild-Type Advanced Melanoma Treated With Nivolumab Therapy: Three-Year Follow-up of a Randomized Phase 3 Trial. *JAMA Oncology.* 2019;5(2):187-194.

26. Bergmann L, Albiges L, Ahrens M, et al. Prospective randomized phase-II trial of ipilimumab/nivolumab versus standard of care in non-clear cell renal cell cancer - results of the SUNNIFORECAST trial. *Annals of oncology : official journal of the European Society for Medical Oncology.* 2025;36(7):796-806.

27. Zandberg DP, Allred JB, Rosenberg AJ, et al. Phase II (Alliance A091802) Randomized Trial of Avelumab Plus Cetuximab Versus Avelumab Alone in Advanced Cutaneous Squamous Cell Carcinoma. 2025;43(21):2398-2408.

28. Vasudev NS, Ainsworth G, Brown S, et al. Standard Versus Modified Ipilimumab, in Combination With Nivolumab, in Advanced Renal Cell Carcinoma: A Randomized Phase II Trial (PRISM). 2024;42(3):312-323.

29. Yau T, Galle PR, Decaens T, et al. Nivolumab plus ipilimumab versus lenvatinib or sorafenib as first-line treatment for unresectable hepatocellular carcinoma (CheckMate 9DW): an open-label, randomised, phase 3 trial. *The Lancet.* 2025;405(10492):1851-1864.

30. Motzer RJ, Rini BI, McDermott DF, et al. Nivolumab plus ipilimumab versus sunitinib in first-line treatment for advanced renal cell carcinoma: extended follow-up of efficacy and safety results from a randomised, controlled, phase 3 trial. *The Lancet Oncology.* 2019;20(10):1370-1385.
